# Fused deposition modeling three-dimensional printing of flexible polyurethane intravaginal rings with controlled tunable release profiles for multiple active drugs

**DOI:** 10.1007/s13346-022-01133-6

**Published:** 2022-02-24

**Authors:** Yufei Chen, Yannick L. Traore, Lyndon Walker, Sidi Yang, Emmanuel A. Ho

**Affiliations:** 1 Laboratory for Drug Delivery and Biomaterials, School of Pharmacy, University of Waterloo, 10A Victoria St. S, Ontario N2G 1C5 Kitchener, Canada; 2 College of Pharmacy, University of Manitoba, Winnipeg, MB Canada

**Keywords:** 3D-printing, Intravaginal ring, HIV, Nanoparticles, Fused deposition modeling

## Abstract

We designed and engineered novel intravaginal ring (IVR) medical devices via fused deposition modeling (FDM) three-dimensional (3D) printing for controlled delivery of hydroxychloroquine, IgG, gp120 fragment (encompassing the CD4 binding site), and coumarin 6 PLGA-PEG nanoparticles (C6NP). The hydrophilic polyurethanes were utilized to 3D-print reservoir-type IVRs containing a tunable release controlling membrane (RCM) with varying thickness and adaptable micro porous structures (by altering the printing patterns and interior fill densities) for controlled sustained drug delivery over 14 days. FDM 3D printing of IVRs were optimized and implemented using a lab-developed Cartesian 3D printer. The structures were investigated by scanning electron microscopy (SEM) imaging and in vitro release was performed using 5 mL of daily-replenished vaginal fluid simulant (pH 4.2). The release kinetics of the IVR segments were tunable with various RCM (outer diameter to inner diameter ratio ranging from 1.12 to 2.61) produced from FDM 3D printing by controlling the printing perimeter to provide daily zero-order release of HCQ ranging from 23.54 ± 3.54 to 261.09 ± 32.49 µg/mL/day. IgG, gp120 fragment, and C6NP release rates demonstrated pattern and in-fill density-dependent characteristics. The current study demonstrated the utility of FDM 3D printing to rapidly fabricate complex micro-structures for tunable and sustained delivery of a variety of compounds including HCQ, IgG, gp120 fragment, and C6NP from IVRs in a controlled manner.

**Graphical abstract:**

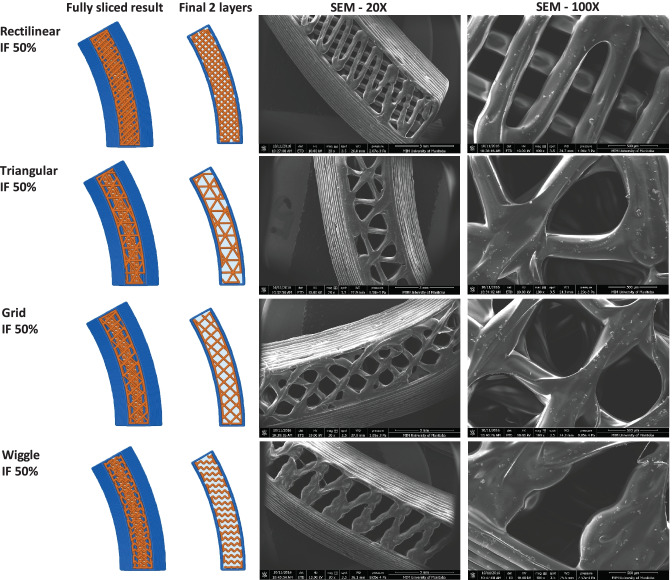

## Introduction

To prevent HIV viral entry and replication, the development of a safe and effective HIV-1 vaccine would undoubtedly be the best solution for the ultimate control of the worldwide AIDS pandemic [1]. Due to the challenges of the extraordinary diversity of HIV-1 [2], the capacity of the virus to evade adaptive immune responses [3], the difficulty to induce broadly reactive antibody responses [4], and the early establishment of latent viral reservoirs [5], HIV-1 vaccine development efforts have not yet proven successful [6]. However, several other strategies attempting to prevent sexual transmission of HIV have been proposed or examined, including the following: (1) pre-exposure prophylaxis (PrEP) via tenofovir disoproxyl fumarate-based regimen as recently recommended by WHO [7]; (2) induction of immune quiescence (IQ) to establish local mucosal resistance to HIV-1 infection by maintaining a low baseline of immune activation and minimizing the HIV-target cells or substrates for productive HIV infection [8–10]; (3) direct delivery of potent neutralizing antibodies (NAbs) topically or induce vaginal mucosal immunity by local mucosal immunization via HIV antigens; (4) topical application of antiviral compounds (microbicides) at the genital tract [11]. IQ model of protection from HIV infection was proposed based on the evidence observed in HIV-seronegative individuals with lowered level of inflammation related cytokines and chemokines production at the female genital tract (FGT), reduced availability of activated CD4 + HIV target cells, and decreased infiltration of HIV target cells to fuel viral propagation and dissemination of HIV infection to the systemic level. This provided the rationale of delivering the immunomodulatory compound hydroxychloroquine (HCQ) locally [8] to maintain low baseline of immune activation at the FGT as well as the inhibition in gene expression of host factors related to viral binding/entry including CCR5 and CD4 by nanoparticle-mediated gene knockdown [12–14]. In addition, potent NAbs can prevent HIV infection by interfering with virus-host cell interactions [15] or may mediate HIV virion clearance by other effector cells (i.e. macrophages, CD8^+^ T cells) via coating the viral particles [16]. Several pre-clinical studies have demonstrated that significantly high levels of antigen-specific antibodies could be elicited using intravaginal immunization [17–19]. Broadly reactive NAb activity has been identified in a small number of HIV-1 infected subjects. This reactivity seems to be largely directed against conserved regions of the viral envelope protein such as the CD4-binding site on gp120 [20]. Utilizing 50 µg of a synthetic peptide encompassing part of the CD4-binding site (spanning the envelope residues 421–438) on the gp120 molecule to subcutaneously immunize mice, rabbit, and goat, it was found that broadly reactive anti-HIV responses against multiple strains of HIV were elicited, resulting in high titers of anti-gp120 antibodies [21]. In the current study, we utilized fused deposition modeling (FDM) three-dimensional (3D) printing to develop different reservoir-type intravaginal ring (IVR) devices for controlled and prolonged delivery of HCQ, IgG, gp120 fragment (containing CD4 binding site), and coumarin 6-loaded poly(lactic-*co*-glycolic acid) (PLGA) nanoparticles.

In 2015, the US Food and Drug Administration (FDA) approved the first 3D printed tablet Spritam, indicating a new era of introducing 3D printing into pharmaceutical dosage forms and medical device development [22–24]. As a layer-by-layer additive process to produce 3D geometries with digitally pre-designed structures generated from the computer-aided design (CAD) software, fused deposition modeling FDM 3D printing is a cost-effective extrusion-based additive manufacturing (AM) technology that mainly utilizes thermoplastic materials for creating solid objects [22, 25]. It offers a versatile manufacturing option to rapidly develop pharmaceutical dosage forms at relatively low cost using the combination of pharmaceutical-grade thermoplastic polymers and active pharmaceutical ingredients (APIs) via the hot-melt extrusion (HME) process to achieve designed release kinetics of different APIs [26, 27]. Generally, these biocompatible polymers are mixed with APIs to form drug-containing filaments via regular HME and the resulting filaments are loaded into FDM 3D printers for producing the designed shapes with drugs dispersed within their matrices. Emerging attempts of developing pre-clinical pharmaceutical dosage forms via FDM 3D printing have been reported for patient-tailored treatment [28–30] along with the development of drug delivery systems that can provide controlled immediate or sustained drug release profiles [31–34]. In addition, personalized topical drug delivery strategies coupled with 3D scanning have also been reported [35]. Various thermoplastic polymers have been explored to be employed in FDM 3D printing to fabricate tablets, capsules, or implants, including both biodegradable and non-biodegradable polymers such as polylactic acid (PLA) [27], polycaprolactone (PCL) [36], polyvinyl alcohol (PVA) [28–30, 37], hydroxypropyl cellulose (HPC) [33], ethylene vinyl acetate (EVA) [38], and poly methyl methacrylate (PMMA) [39]. During FDM 3D printing, several controllable variables affect the printing quality and contribute to the final geometry formation including thickness of each layer, gap width and angle between layers, infill density, infill pattern, extrusion speed, and nozzle temperature [40]. In comparing to the traditional HME methods, FDM 3D printing possesses several distinct advantages such as reduced cost in equipment configuration, operation and maintenance, the capability of rapid evaluation of various polymers with considerably low material and time consumption, the ease of switching multiple polymers during a single test, and most appealing, the ability to fine-tune control of drug release rates via the alteration of printing geometries at mesoscale level, which is impossible to achieve using traditional HME or hot injection molding processes limited by the designs of dies or molds. Control of FDM 3D printing parameters can produce desired pore size, interconnectivity and morphology on the printed objects [41]. Moreover, the ability to form pre-designed hollow structures via FDM 3D printing enables the possibility of tuning the drug release profiles [33, 34, 42]. It was demonstrated that water soluble API release rates from hollow implants printed with drug-containing polymeric filament was predominately determined by the polymer utilized and proportional with drug loading in the filament [34]. To the best of our knowledge, our research team is the first to evaluate the potential of applying FDM 3D printing to generate drug-eluting reservoir-type IVR devices by investigating the effects of print pattern and interior fill (IF) density on drug release kinetics.

## Materials and methods

### Materials

Hydroxychloroquine sulfate (HCQ, purity > 98%) was purchased from ThermoFisher. Medical-grade non-biodegradable hydrophilic thermoplastic polyurethanes HP-60D-35 (35% water adsorption) and ATPU-75A (~ 1.29% water adsorption) were obtained from Lubrizol Corporation and DSM, respectively. Polyvinyl alcohol (PVA) (31 ~ 50 KDa) and Coumarin 6 (C6, 3-(2-benzothiazolyl)-*N,N*-diethylumbelliferylamine, 3-(2-benzothiazolyl)-7-(diethylamino) coumarin) were purchased from Sigma-Aldrich (ON, Canada). Carboxylic acid terminated PLGA (50/50)(10 KDa)-PEG(2 KDa) was synthesized by Advanced Polymer Materials (QC, Canada). The synthetic fragment of gp120 molecule containing the conserved CD4-binding domain (residue 421–438, sequence: KQFINMWQEVGKAMYAPP, 2,138.56 Da) was purchased from Biomatik. Reference human immunoglobulin G (IgG) antibody (4.4 mg/mL) was purchased from Cedarlane Laboratories Ltd. (Burlington, Ontario, Canada). Low viscosity hydroxypropyl methylcellulose (HPMC), METHOCEL™ E4M (assayed apparent viscosity: 12–14 mPa⋅s), was kindly provided by The Dow Chemical Company (Calgary, Alberta, Canada). Human IgG ELISA kit was purchased from Bethyl Laboratories Inc. (Montgomery, TX, USA). HPLC-grade trifluoroacetic acid (TFA), acetonitrile, methylene chloride, methanol, and heptanesulphonic acid were purchased from VWR International LLC (Batavia, IL, USA). Dyna-purge^®^ M polymer was obtained from Shuman Plastics (Buffalo, NY, USA).

### FDM 3D printing of IVR segments

**Fig. 1:**
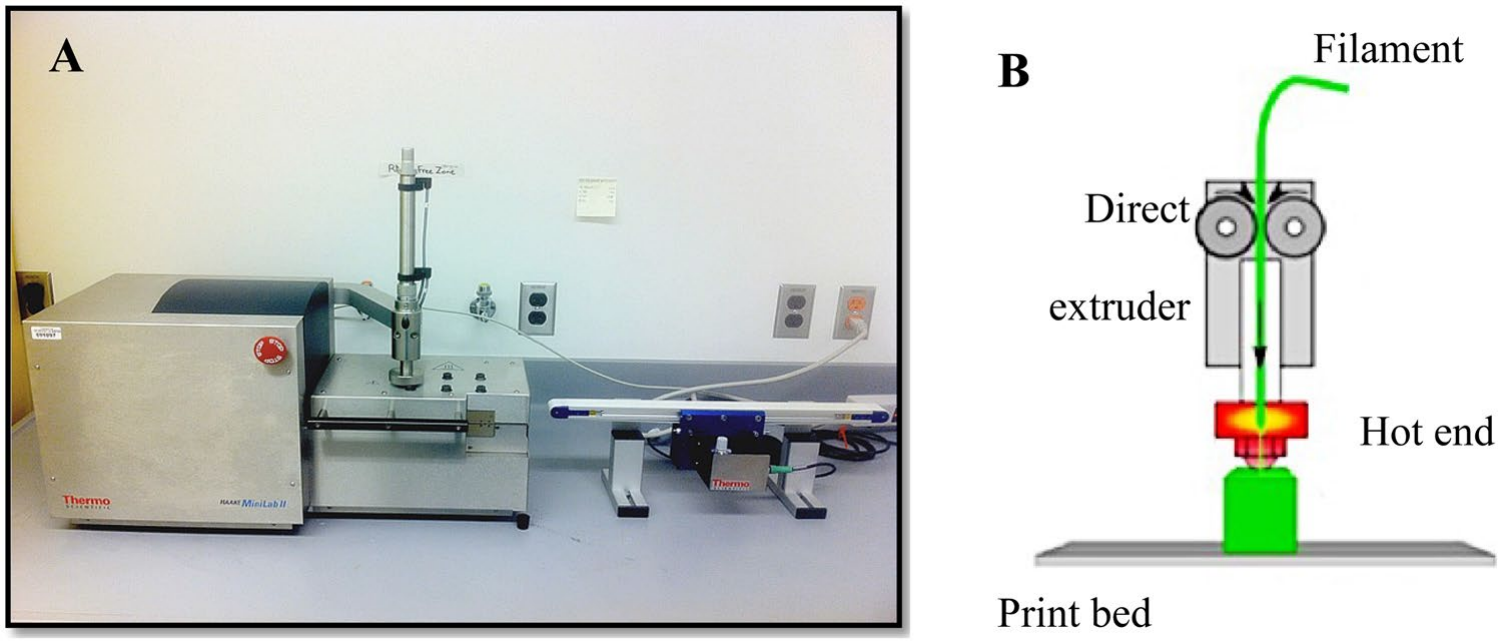
Hot-melt extruder and FDM 3D printer used in current study. **a**HAAKE™ MiniLab II Micro Compounder utilized for fabricating medical-grade polyurethane filament for FDM 3D printing. **b** Schematic illustration of the printing process involving some of the key components of lab-developed FDM 3D printer utilized in the present study

HP-60D-35 and ATPU-75A pellets were completely dried at 80 °C overnight to remove any moisture. The dried pellets were then extruded to form flexible filaments with an outer diameter of 1.6 ± 0.05 mm via a 2-mm rod die attached to a HAAKE™ MiniLab II Micro Compounder (ThermoFisher) according to manufacturer’s recommended extrusion temperatures (160 °C and 150 °C, respectively). Continuous filament extrusion was performed by coupling the MiniLab II to a conveyer belt system (Fig. 1A). Filament diameter was regularly measured using a digital caliper during extrusion. Each fabricated filament was then placed in an oven (SVAC1E SHEL LAB Economy Vacuum Oven, Sheldon Manufacturing, Inc., Cornelius, OR, USA) pre-heated up to 40 °C during printing to protect it from absorbing atmosphere moisture. IVRs (macaque size: 25 mm outer diameter and 5.5 mm cross-sectional diameter) and IVR segments (1/4 of a full-size macaque IVR) were digitally designed in Geomagic Design 2015 (3DS Systems) and exported as stl files. A lab-developed Cartesian FDM 3D printer (Fig. 1B), containing a direct extruder for hot extrusion of the fabricated filament, was employed for performing the printing. Simplify3D v3.1 (Simplify3D, LLC) was utilized for both slicing the designed 3D models and controlling the printer. For reservoir IVR HCQ release studies, HP-60D-35 filament was fed into the 3D printer and printed with different RCM (rate controlling membrane). Hot end was set at 225 °C (cooling fan on) with 210 µm nozzle extrusion width. Printing was conducted at a printing speed of 15 mm/s at 100 µm layer height. Other printing parameters were set as follows: X/Y axis movement at 300 mm/s; Z axis movement at 100 mm/s; outline overlap at 1%; infill (IF) extrusion width at 100%; minimum IF length at 10 µm; extrusion multiplier set at 1. Different perimeter shells with 0% infill were chosen in slice settings to produce macaque-sized IVRs with different ro/ri ratios (outer radius to inner radius ratios). IVR segments designed for C6 PLGA-PEG nanoparticle (C6NP), gp120 fragment and IgG release, were printed using ATPU-75A filaments at similar conditions but at a higher printing temperature of 220 °C. The final two layers of the segment was programmed in Simplify3D to be sliced with desired IF and printing patterns to form different geometries. Retraction related parameters were disabled to avoid any retraction during printing due to the high flexibility of both HP-60D-35 and ATPU-75A filaments. To investigate the effects of different IF and printing patterns on C6NP and protein release, different IF ranging from 25 to 100% and IF patterns (including rectilinear, triangular, grid, wiggle, fast honeycomb, full honeycomb) were printed. Fast honeycomb refers to a smaller honeycomb structure while full honeycomb refers to a larger honeycomb structure.

### Preparation of release rate-controlling semi-solids containing HCQ, IgG, and gp120 fragment

HPMC semi-solids containing HCQ, IgG, and gp120 fragment were prepared as previously described [43] as a means to control the release of the APIs. Briefly, to prepare semi-solid with desired weight ratio of HPMC to HCQ (1:1 wt ratio), the appropriate amount of HPMC powder was first weighed into a 10-mL beaker. HCQ solution (200 mg/mL) was added in a step-wise fashion and mixed thoroughly with HPMC powder simultaneously. After that, all the content was then transferred into a 3-mL syringe attached to another 3-mL syringe via a 1.5 cm length of polypropylene tubing. The HCQ/HPMC semi-solid was further mixed thoroughly but gently by passing the samples from one syringe to another for a minimum of 100 passages. HPMC semi-solids containing IgG and gp120 fragment were prepared similarly using concentrated IgG and gp120 fragment solutions at 4.4 mg/mL and 4 mg/mL, respectively. Uniformity of HCQ, IgG, and gp120 fragment dispersion within the semi-solid was determined by randomly sampling and extracting the API from 10 ± 0.5 mg of semi-solid using 1 mL of water, incubated overnight at 4 °C, and quantitated using HPLC.

### Preparation of C6-PLGA-PEG nanoparticles

C6 was encapsulated into PLGA-PEG NP by a double-emulsion evaporation method using the biodegradable di-block copolymer, PLGA-PEG [44]. In brief, PLGA-PEG (10 mg/mL) was dissolved in methylene chloride and equal volumes (600 µL) of C6 solution (40 mg/mL) was then continuously emulsified with of PLGA-PEG solution for 15 s. The primary emulsion was further emulsified with 4.3 mL of 2% PVA for 3 min, forming a w/o/w emulsions. The resulting emulsion was stirred overnight at 4 °C to evaporate the organic solvent and to harden the NP. NP was then collected by centrifuge (20,000 g for 15 min at 4 °C) and washed twice with water to eliminate excess PVA and unencapsulated C6. The resulting C6NP was resuspended in water and nanoparticle size, surface zeta-potential, and polydispersity index (*PDI*) was determined using a Brookhaven ZetaPALS Potential Analyzer (Brookhaven Instruments Corporation, NY, USA).

### Scanning electron microscopy

Images of 3D printed IVR segments were taken using a FEI quanta 650 SEM (FEI Corporate, Oregon, USA) under low vacuum mode at the Manitoba Institute for Materials. FEI Quanta 650 is a variable pressure field emission SEM for high resolution images.

### In vitro release studies

3D-printed HP-60D-35 segments with different RCM thickness were prepared by loading HPMC semi-solid containing 20 mg of HCQ and drug release was evaluated over 14 days. Ends of IVR segments were tightly sealed with non-permeable polyurethane to ensure that HCQ only diffuses across the RCM. Different RCMs were defined by different ratios of outer radius (ro) to inner radius (ri), ranging from 1.12 to 2.61, reflecting 1 to 7 3D printed perimeter shells. The ro of the macaque-sized IVR is 5.5 mm while the ri was calculated by subtracting 2 times of the measured RCM, resulting in the ri values required for final calculations of ro/ri ratios. The “capped” segments were then incubated in 5 mL of vaginal fluid simulant (VFS, pH 4.2) in 20-mL scintillation vials in an incubating orbital shaker set to 37 °C and 100 rpm. After collecting the sample every 24 h, 5 mL of fresh VFS was replenished. HCQ concentrations in collected samples were quantitated using the RP-HPLC method reported previously [45, 46]. Briefly, Waters Nova-Pak^®^ C18 column (4 µm, 3.9 × 150 mm) was used under an isocratic condition in the Shimadzu LC-2010A HPLC system. The mobile phase (pH 3.1) consisted of methanol, acetonitrile, and 58 mM sodium phosphate dibasic buffer (4:22:74, v/v) with 6 mM heptanesulphonic acid. Flow rate was 1.0 mL/min and the UV detection was set at 343 nm. Forty microliters of samples were injected into the Waters^®^ Alliance^®^ HPLC system equipped with Waters^®^ 2690 Separations module and Waters^®^ 996 Photodiode Array detector for analysis.

Similarly, HPMC semi-solids containing 2 mg of IgG and gp120 fragment were loaded into the lumen of printed ATPU-75A segments that were end-capped in the same way. Semi-solid containing segments were incubated in 5 mL of VFS (without BSA to interfere with quantitation methods for IgG and gp120 fragment) inside 20-mL scintillation vials in the shaker at 37 °C and 100 rpm. Samples were collected at intervals of 24 h for 14 days and stored at −20 °C until further analysis. One hundred microliters of sample was subjected to a human ELISA kit (Bethyl Laboratories Inc.) for quantitating the released IgG in VFS with a lower limit of quantitation (LLOQ) of 0.69 ng/mL. For determining the levels of gp120 fragment in the samples, a previously developed gradient RP-HLPC method was utilized [45]. Briefly, analysis was performed using a XBridge™ BEH300 C18 column (300 Å, 5 µm 4.6 × 150 mm; Waters) with a Symmetry C18 guard column (300 Å, 5 µm, 3.9 × 20 mm; Waters), fitted to a Waters^®^ Alliance^®^ HPLC system equipped with Waters® 2690 Separations module and Waters^®^ 996 Photodiode Array detector. Mobile phase A consisted of 0.1% TFA in 10% acetonitrile in HPLC grade water. Mobile phase B consisted of 0.85% TFA in 90 acetonitrile in water. A gradient A/B was applied to elute and analyze the gp120 peptide: A/B from 85:15 to 20:80 in 6 min, followed by maintaining at 20:80 for 2 min and re-equilibration of the column at 85:15. Total run was 10 min for each 200 μL injection. Flow rate was 2.0 mL/min, UV detection was set at 210 nm, and column temperature was maintained at 60 °C. The retention time for gp120 was approximately 2.7 min. The linear calibration curves obtained for gp120 were in the range of 0.625–80 µg/mL (*R*^2^ > 0.999) with a LLOQ of 0.31 μg/mL.

For C6NP in vitro release studies, 160 μg of C6NP in water without any excipients were added into printed ATPU-75A IVR segments for a 14-day release under the same condition as described above. Five milliliters of VFS was used as release medium and replaced daily after sample collection. To quantitate the C6NP levels, 200 μL of samples were analyzed using a multiplate reader (BioTek Synergy™) with an excitation wavelength of 485 nm and emission wavelength of 528 nm to determine its fluorescence intensity. The linear standard curve of C6NP was obtained in the range of 0.625–40 µg/mL (*R*^2^ > 0.999) with the LLOQ at 0.625 μg/mL.

### In vitro cytotoxicity studies

#### Cell culture

In vitro cytotoxicity studies were performed using the human vaginal epithelial cell line VK2/E6E7, ectocervical epithelial cell line Ect1/E6E7, and human T cell line SupT1 (ATCC, Rockville, MD, USA). The vaginal and ectocervical epithelial cells were cultured in keratinocyte-serum free medium (K-SFM) with 0.1 ng/mL of recombinant human epidermal growth factor, 50 mg/mL of bovine pituitary extract (Life Technologies, Carlsbad, CA, USA), 0.4 mM CaCl2 (VWR International, Batavia, IL, USA), and 1% penicillin/streptomycin (Sigma-Aldrich). SupT1 cells were maintained in RPMI-1640 medium supplemented with heat-inactivated fetal bovine serum. All cells were cultured at 37 °C and 5% CO2 prior to the experiments.

#### Elution assay

The effect of 3D printed IVR segments on cell viability was investigated using the elution assay described previously [43, 46]. Briefly, after FDM printing, the resulting hollow HP-60D-35 and ATPU-75A segments were sterilized by immersion in 70% alcohol for 30 s inside a sterile biosafety cabinet. Once air dried, each segment was incubated in 10 mL of either complete K-SFM medium or RPMI-1640 medium in a sterile 15 mL tube using aseptic technique. The samples were incubated at 37 °C for 1, 7, 15, and 30 days in an orbital shaker at 100 rpm. At each interval, the resulting elution medium from the printed segments was stored at −80 °C until further analysis.

To determine the effect of collected elution medium on cell viability, CellTiter 96^®^ AQueous One Solution Cell Proliferation Assay (MTS assay, Promega Corporation, WI, USA) was conducted by incubating collected medium with freshly seeded cells (at 2.5 × 10^4^ per 100 µL per well in a 96-well plate) for 24 h. Twenty microliters of MTS assay reagent was added to each well and absorbance of each well was measured at 490 nm. In addition, the resulting supernatants were collected and the concentrations of pro-inflammatory cytokines and chemokines (IL-1ß, IL-6, and IL-8) were determined using sandwich ELISA kits from R&D Systems (ON, Canada).

### Statistical analysis

Data are presented as mean ± standard deviation (SD). The *N* value refers to number of replicates performed for each experiment. Student’s *t* test (unpaired, two-sample, unequal variance with two-tailed distribution) was performed. Multiple comparisons was performed using one-way ANOVA coupled with Bonferroni comparison as post-test using GraphPad Prism 6.0 (GraphPad Software, Inc., La Jolla, CA, USA) with *p* < 0.05 considered significant difference.

## Results

### 3D-printed polyurethane IVRs

**Fig. 2:**
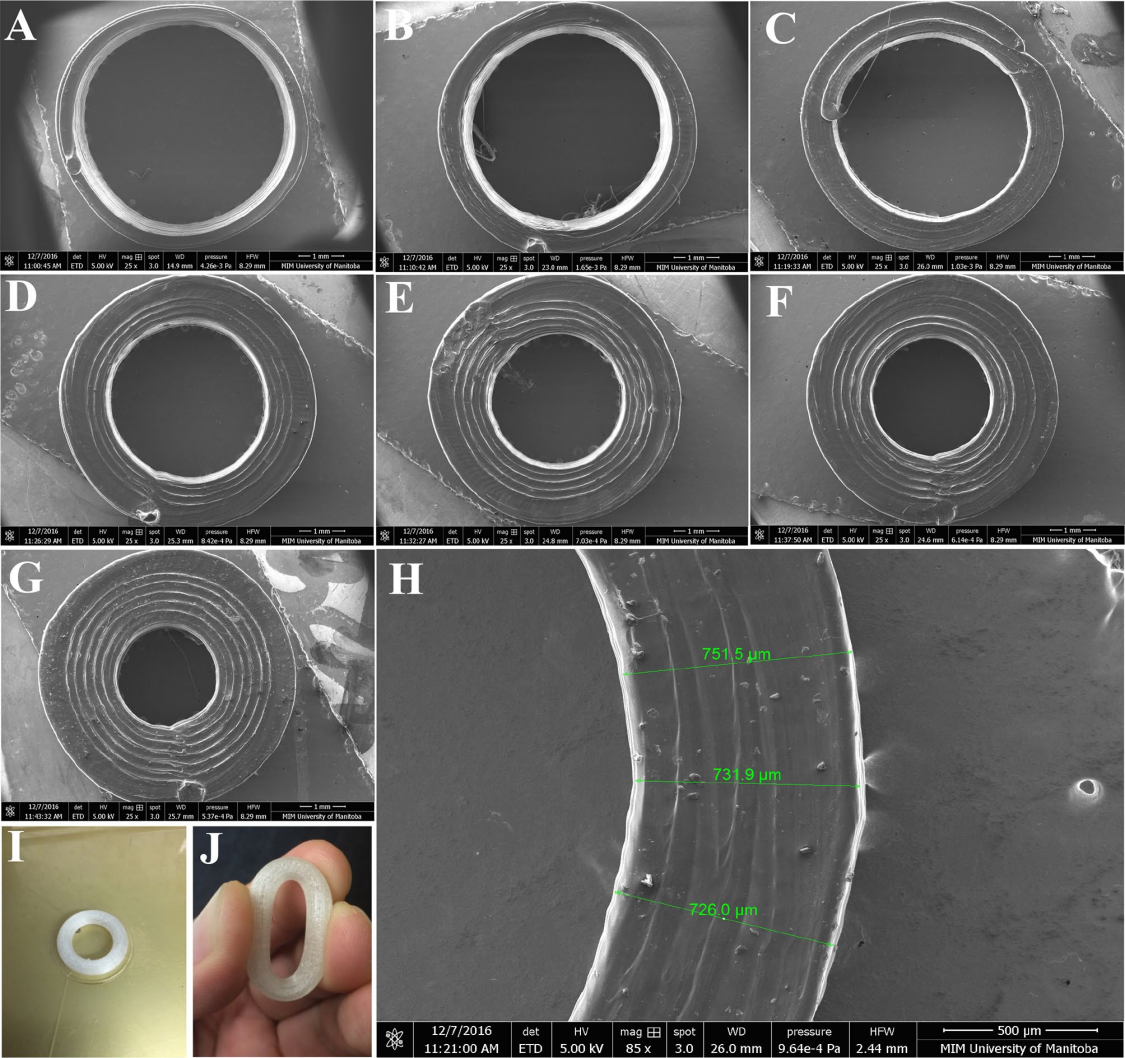
SEM imaging of FDM 3D printed HP-60D-35 segments and macaque-size IVR. Panels (**A**–**G**) demonstrated the printed segment with different perimeter shells incrementally from 1 to 7 shells (ro/ri ratios ranging from 1.12 to 2.61). Panel (**H**) depicts an example RCM thickness measurement of a printed segment with 3 shells. A full-size macaque reservoir-IVR can be directly printed as shown in panel (**I**) with adequate flexibility demonstrated in panel (**J**)

**Fig. 3:**
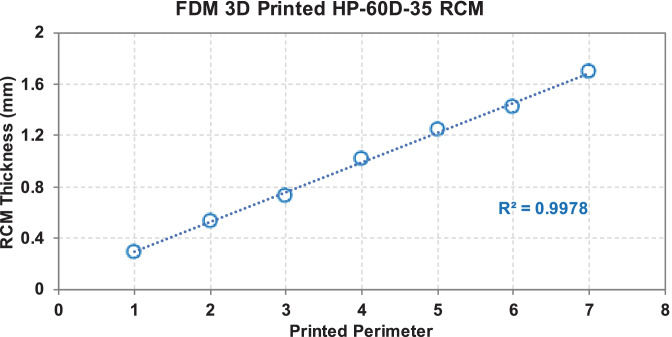
Correlation of RCM thickness and printed perimeter. A linear relationship between the measured RCM thickness and the selected printing perimeter was observed

Using the described printing condition, full-size macaque reservoir-type HP-60D-35 IVR and IVR segments (10 mm in length) with different RCM can be directly printed as shown in Fig. 2. RCM can be easily tuned by changing the perimeter settings to obtain different thickness. In the current study, a range of 0.30 to 1.69 mm of RCM can be achieved by increasing the printing perimeter to 7 shells at an increment of 1 shell, resulting in ro/ri ratios from 1.12 to 2.61, respectively, as shown in Table 1. SEM images of printed segments with 1 to 7 perimeters are shown in Fig. 2 panel A to G. The increased RCM thickness is proportional to the printed perimeters selected (Fig. 3).

**Table 1:** SEM measurements of printed HP-60D-35 segments and their corresponding outer radius/inner radius (ro/ri) ratios

**Perimeter shell (P)**	**RCM measured by SEM (mm)**	**SD**	**ro/ri ratio**	**Representative segment shown in Fig. **2
1	0.30	0.01	1.12	A
2	0.54	0.04	1.24	B
3	0.73	0.01	1.36	C
4	1.02	0.02	1.59	D
5	1.25	0.02	1.83	E
6	1.42	0.02	2.07	F
7	1.69	0.03	2.61	G

*RCM* rate controlling membrane, *SEM* scanning electron microscopy, *SD* standard deviation

Data was obtained from 4 individually printed segments at each printing perimeter. Measured RCM was expressed as mean values with SD calculated, *n* = 4

**Fig. 4:**
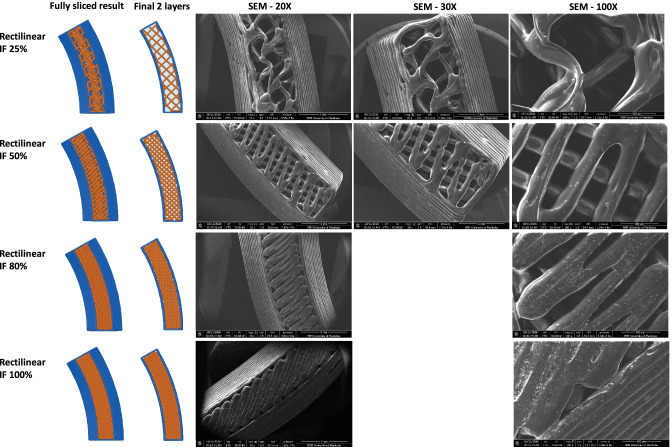
Comparison of ATPU-75A segments printed at different IF using rectilinear pattern. The stl file of the segment was sliced using Simplify3D. The complete sliced results are shown along with the sliced final two layers. SEM images were obtained at 20X, 30X, and 100X. Note: there were no 30X images captured for the Rectilinear IF 80% and Rectilinear IF 100% groups

**Fig. 5:**
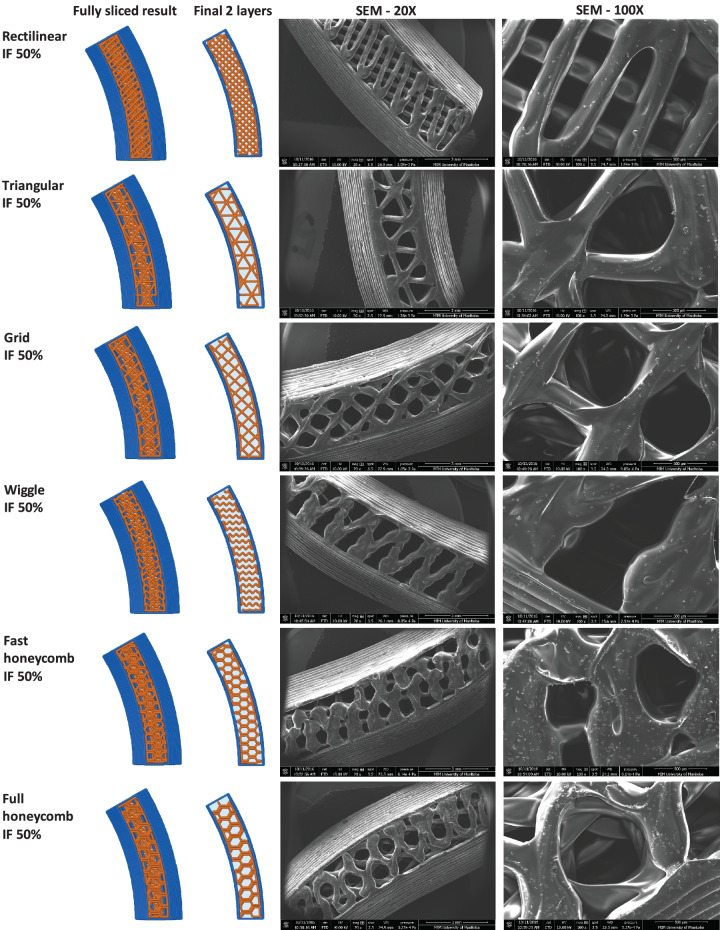
Comparison of ATPU-75A segments printed at 50% IF using different patterns

**Fig. 6:**
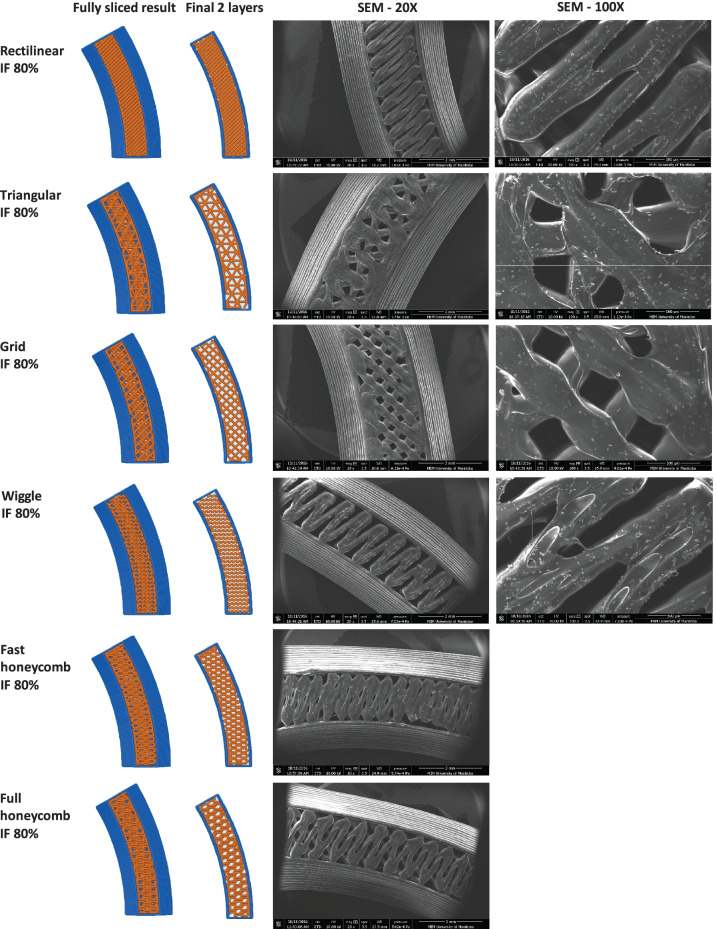
Comparison of ATPU-75A segments printed at 80% IF using different patterns

Since another focus of the current study was to investigate the effect of IF density and printing patterns on the protein and C6NP release kinetics, one side of the reservoir ATPU-75A IVR segments were printed with different IF densities and printing patterns (Figs. 4-6). First, we compared ATPU-75A segments printed at different IF under the same rectilinear pattern to investigate the effect of IF on porosity (Fig. 4). Less porosity was observed as IF increased from 25 to 100%. An IF of 50% resulted in the formation of a surface consisting of approximately 250 μm pores. The printed segments showed IF and printing patterns similar to the programmed slicing outcomes. However, a less desired printing result was seen at 25% IF possibly attributed to the rapid XY movement of the print head resulting in insufficient filament extrusion and adherence to the previous layer under such speed. As expected, IF at 100% showed nearly no distinct porosity difference regardless of the printing patterns selected (data not shown). Therefore, 50% and 80% IF were chosen for further assessment. Both the 50% IF printing (Fig. 5) and 80% IF printing (Fig. 6) returned a distinct porous structure formed on the segment and demonstrated high similarity to the slicing results generated by Simplify3D. Of all the patterns evaluated that were able to form porous structures with high similarity to the slicing results, triangular pattern showed the largest pore sizes formed (> 500 μm), followed by grid pattern (500 μm), and rectilinear pattern (250 μm). Wiggle pattern formed triangular porous structures while fast honeycomb and full honeycomb patterns formed irregular pores on the surface. At 80% IF, only segments printed in triangular and grid patterns showed similar printing outcomes as the slicing results, forming 150–200 μm pores. Segments printed in grid pattern demonstrated a more uniformed porous surface in comparison to those printed in triangular pattern. All other printing patterns showed no distinct differences at 80% in comparison to those printed with rectilinear pattern, indicating the limit of XY resolution using the current printer was approximately 200–250 μm.

### Modulation of HCQ release rates by printing perimeter

**Fig. 7:**
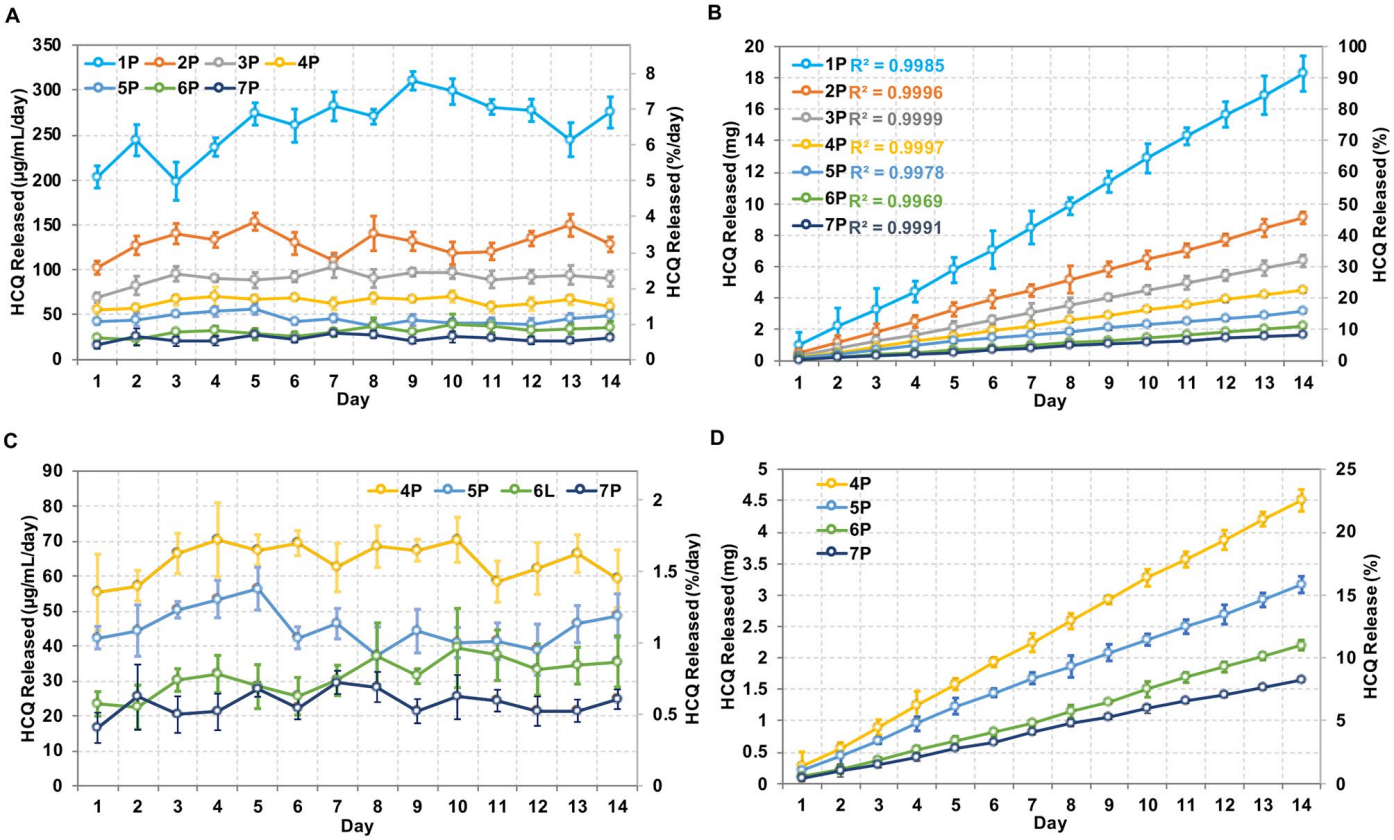
In vitro release of HCQ from printed HP-60D-35 segments. (**A**) In vitro daily release and (**B**) cumulative release of HCQ from 3D printed segments with different printed perimeter shells during 14-day study period. (**C**) and (**D**) were plotted separately to show HCQ release from segments printed at 4 perimeter shells and above, containing thicker RCM and lower release rates. 1P–7P, segments printed with 1 to 7 perimeter shells. Data expressed as mean ± SD, *n* = 4

Since drug diffusion from a cylinder reservoir-type delivery system fabricated with drug diffusible polymers is predominantly determined by the thickness of RCM [47, 48], we compared the HCQ release rates from HP-60D-35 segments printed with different perimeters (Fig. 7). The 2-week in vitro HCQ release kinetics from segments was correlated with different printing perimeters resulting in various RCM thickness. HCQ elution from the printed segments with distinct RCM thickness demonstrated zero-order release profiles ranging from 23.54 ± 3.54 to 261.09 ± 32.49 µg/mL/day (Fig. 7A and C), which were all above the clinically effective blood concentration of HCQ required to elicit anti-inflammatory and T-cell immune quiescent activity [49]. The cumulative release of HCQ (Fig. 7B and D) ranged from 18.27 ± 0.16 to 0.58 ± 0.08 mg from the segments printed with 1 perimeter (0.30 mm in RCM thickness) to those printed with 7 perimeters (1.69 mm in RCM thickness). The segments with the thinnest RCM showed the fastest HCQ elution while the drug elution rates became less distinct but still tunable once printing perimeters were above 3 (RCM thickness > 0.73 mm). The segments printed with 1 to 7 perimeters released 91.38 ± 5.63%, 45.40 ± 1.87%, 31.80 ± 1.89%, 22.52 ± 0.86%, 15.83 ± 0.65%, 11.01 ± 0.40%, 8.24 ± 0.15% of total loaded HCQ over 14 days, respectively. Overall, the results indicate that fine-tunable release of HCQ can be easily achieved by changing the printing perimeters, which was determined by the RCM thickness. Prolonged zero-order release over 14 days can be achieved with increased printing perimeters.

### Sustained release of IgG and gp120 fragment altered by different pore sizes printed

**Fig. 8:**
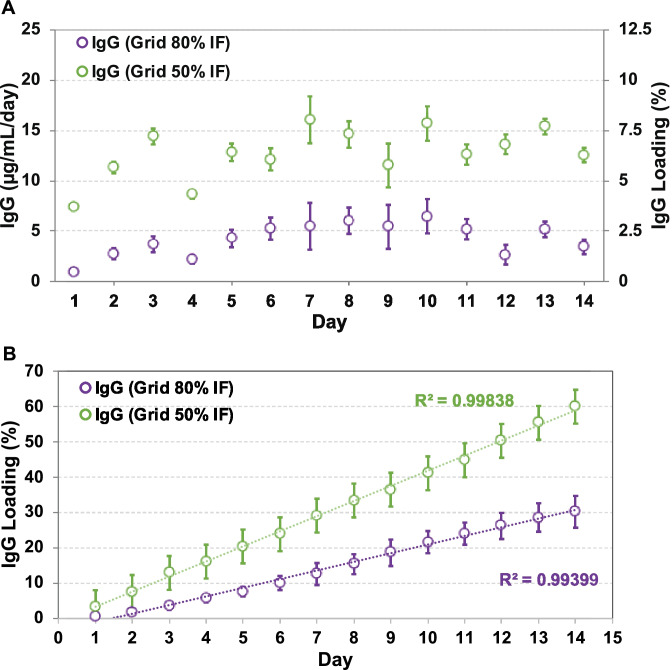
IgG release from printed ATPU-75A segments. (**A**) Daily release and (**B**) cumulative release of IgG from FDM printed ATPU-75A segments in grid pattern with 50% and 80% IF. Data expressed as mean ± SD, *n* = 4

**Fig. 9:**
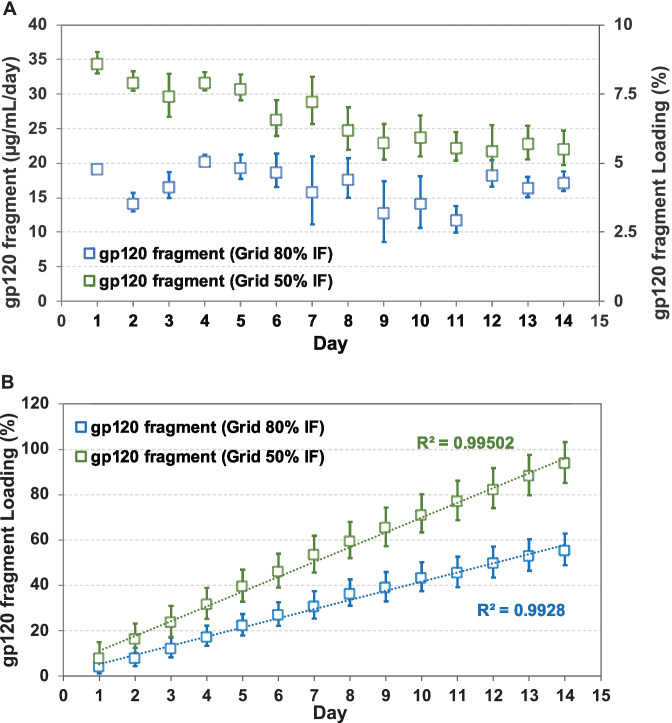
Gp120 fragment release from printed ATPU-75A segments. (**A**) Daily release and (**B**) cumulative release of gp120 fragment from FDM printed ATPU-75A segments in grid pattern with 50% and 80% IF. Data expressed as mean ± SD, *n* = 4

Due to the more uniform porosity formed on the segments printed with the grid pattern and almost twice the size of the pores formed using 50% IF printing (Figs. 5, 6), IVR segments printed in grid pattern at 50% and 80% IF were chosen for IgG (Fig. 8) and gp120 fragment (Fig. 9) release studies. IgG release rates from the printed ATPU-75A segments demonstrated clear correlation with the pore sizes formed on the surface. The release rate of IgG doubled in segments with 50% IF (17.13 ± 3.39 µg/mL/day) in comparison to those printed with 80% IF (8.42 ± 3.27 µg/mL/day). It appeared that IgG diffusion from both segments took 3–4 days to achieve equilibrium and near zero-order diffusion of IgG from both printing IF were seen during the study period (*R*^2^ = 0.99838 and *R*^2^ = 0.99399 for 50% and 80% IF, respectively). During the 14-day study, a total of 1199.09 ± 89.89 µg and 603.54 ± 65.25 µg of IgG were released from segments printed in 50% and 80% IF, respectively.

Pore size printed under different IF regulated gp120 fragment elution from the segment with a similar trend but to a lesser extent. An average release rate of 26.89 ± 4.36 µg/mL/day and 15.71 ± 3.16 µg/mL/day from printed segments with 50% and 80% IF, respectively, was observed for gp120 peptide. Higher release rate was also observed in the segments printed with less IF. A slight burst release on the first day followed by a rapid equilibrated release rate was observed in both segments printed in different IF. Segments printed with both IF showed near zero-order release (*R*^2^ = 0.99502 and *R*^2^ = 0.9928 for 50% and 80% IF, respectively). Within the 14-day study period, noticeably more gp120 fragment was released in comparison to IgG (a total of 1882.21 ± 94.11 µg and 1174.36 ± 65.85 µg of gp120 fragment release from segments printed with 50% and 80% IF, respectively).

### Tunable C6NP release through printing pattern and interior fill density

**Fig. 10:**
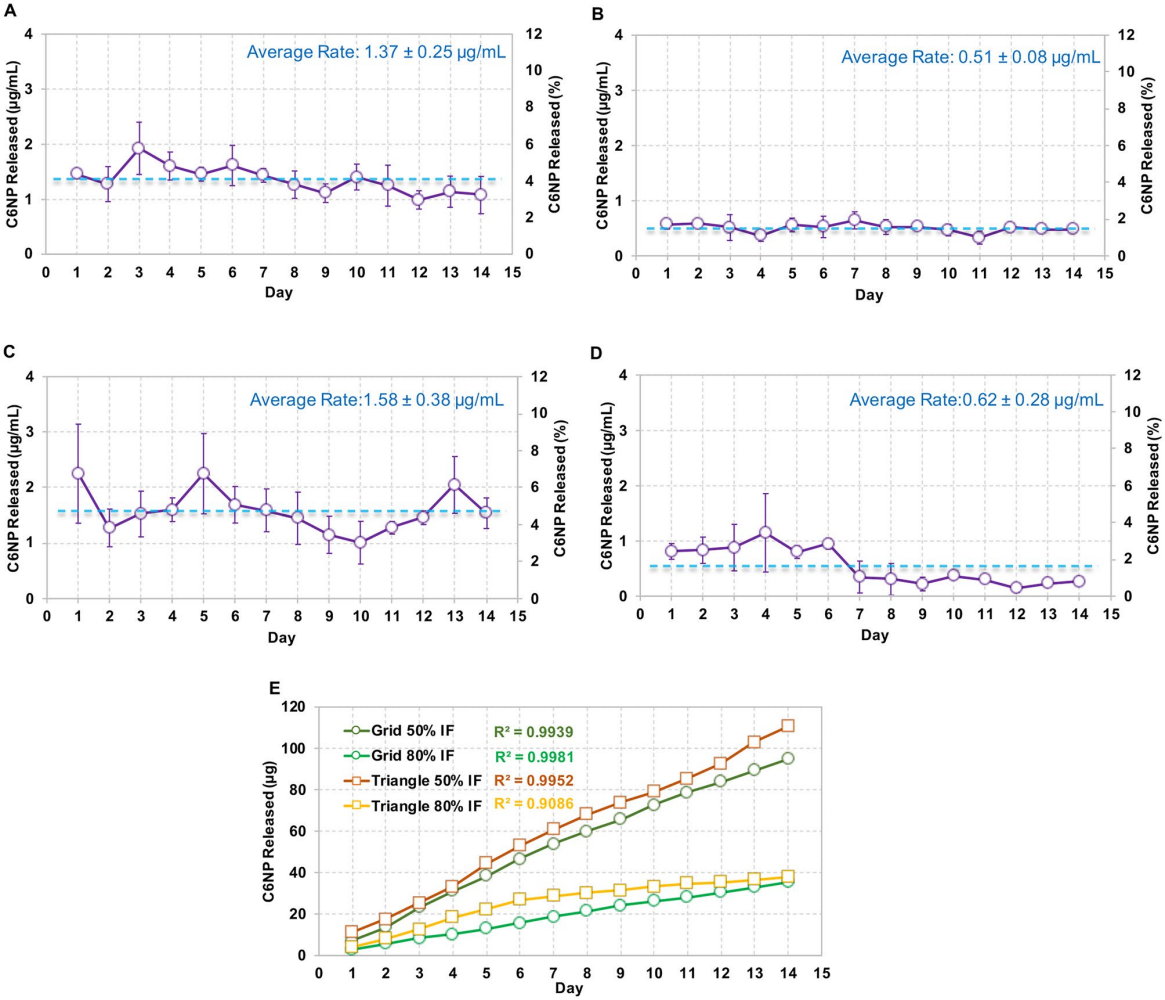
C6NP release from printed ATPU-75A segments. The comparison of C6NP release rates from segments printed in grid pattern at (**A**) 50% IF and (**B**) 80% IF or from segments printed in Triangular pattern at (**C**) 50% IF and (**D**) 80% IF. Blue lines indicated the average release rates over the 14-day study period. (**E**) showed the cumulative release rates from different groups. Data expressed as mean ± SD, *n* = 4

In the current study, the prepared C6NP (particle size at 283 nm, *PDI* = 0.27) was used for the in vitro release study performed in VFS. A total of 160 µg of C6NP was loaded into the lumen of the printed ATPU-75A segments. Segments printed in grid and triangular patterns were selected to investigate the effects of both printing pattern and IF upon PLGA-PEG nanoparticle release (Fig. 10). Grid pattern showed slightly slower daily release rates (1.37 ± 0.25 µg/mL and 0.51 ± 0.08 µg/mL for 50% and 80% IF, respectively) compared to triangular print pattern (1.58 ± 0.38 µg/mL and 0.62 ± 0.28 µg/mL for 50% and 80% IF, respectively). However, a more consistent daily release was maintained from the segments printed in grid pattern. Moreover, both patterns showed increased release rates with 50% IF compared to 80% IF. C6NP release rates increased more than 2 folds with 50% IF for both printing patterns, indicating that IF was the more predominant factor affecting C6NP release rate. The overall cumulative release of C6NP was significantly higher in 50% IF (59.28 ± 0.79% and 69.13 ± 1.19% for grid and triangular patterns, respectively) compared to 80% IF (22.12 ± 2.57% and 23.69 ± 1.05% for grid and triangular patterns, respectively).

### In vitro biocompatibility studies

**Fig. 11:**
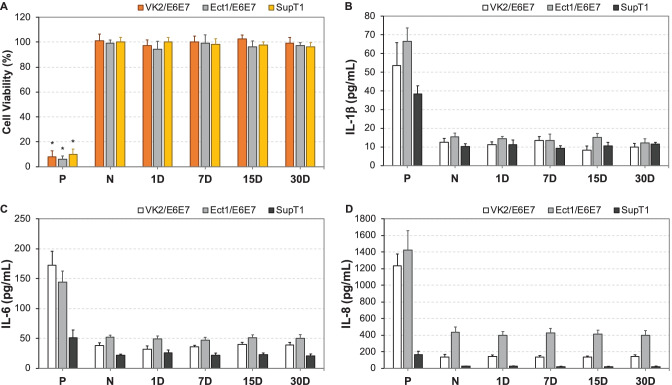
In vitro biocompatibility evaluations of FDM 3D printed ATPU-75A segments. (**A**) MTS assay was performed to determine the cell viability. Data is normalized to the negative control and expressed as mean ± SD, *n* = 4. Cell culture in regular medium was used at negative control. 1 M acrylamide dissolved in normal cell culture medium was used to induce cell death as positive control. (**B**) IL-1β, (**C**) IL-6, and (**D**) IL-8 secretions into the culture medium were determined using sandwich ELISA assays. Plain culture medium was used as negative control. 200 µg/mL of nonoxynol-9 or 50 µg/mL of lipopolysaccharide-treated cells were used as positive controls for IL-1β and IL-6/IL-8, respectively. P, positive control. N, negative control. Data expressed as mean ± SD, *n* = 4. * *p* < *0.05* versus negative control

Intravaginal drug-eluting devices such as IVRs need to demonstrate long-term biocompatibility with the vaginal epithelium [50] without eliciting the production of pro-inflammatory mediators such as IL-1β, IL-6, and IL-8 [51]. Previously, we demonstrated that HP-60D-35 polyurethane was non-cytotoxic to vaginal/ectocervical epithelial and T cell lines and was non-cytotoxic to lactobacilli bacteria commonly found in healthy human female genital tract (FGT) [43, 46]. As a result, we evaluated the impact of FDM 3D printed ATPU-75A segments on cellular cytotoxicity of vaginal/ectocervical epithelial cells and in the CD4 + T cell line SupT1 (Fig. 11). The segments printed with ATPU-75A demonstrated no significant effects on the cell viability of the tested cell lines after incubation for 30 days (Fig. 10A). Similarly, no significant increase in the secretion of IL-1β, IL-6, or IL-8 was observed in the assayed supernatants (Fig. 11B to D). Overall, our FDM 3D printed ATPU-75A segments were non-cytotoxic to human vaginal or cervical epithelial cells and T lymphocytes.

## Discussion

A variety of pharmaceutical-grade polymers with distinct physicochemical characteristics have been explored for developing solid dosage forms or medical devices via the extrusion-based FDM 3D printing process. Commonly, the APIs are co-extruded with the selected polymers to form drug-containing filaments and FDM 3D printing was then applied to transform the filaments into solid objects with desired shapes [29–31, 33, 35, 40]. In these printed dosage forms, the release of APIs is primarily based on passive diffusion regulated by the rate of erosion of the polymeric matrix (affected by matrix weight and composition) [52], swelling properties of the selected polymer [26], or the IF density of the printed matrices such as 3D printed tablets [34]. There are several known advantages of FDM 3D printing such as its compatibility with numerous thermoplastic polymers with different properties to modify the release kinetics of embedded APIs, Unfortunately, there are also several drawbacks observed when fabricating matrix-based drug delivery systems including the following: (1) the time-consuming trial-and-error process for identifying the appropriate polymers needed and the optimal ratio of polymer to API and the (2) inability to incorporate heat-sensitive APIs within the printed dosage form due to the high heat exposure during the HME extrusion and FDM printing process which may result in unwanted degradation of active APIs [35]. For the first time, our research team investigated the feasibility of applying FDM 3D printing to produce mechanically flexible reservoir-type IVR drug delivery systems and evaluated in vitro*,* its potential to fine-tune the release kinetics of hydrophilic API (HCQ) as well as controlling the release rates of heat-sensitive APIs (proteins and PLGA-PEG nanoparticles) via different printing patterns and IF densities.

Medical-grade non-biodegradable polyurethanes have been widely investigated for manufacturing different types of IVRs for contraception [47, 53] or prevention of sexually transmitted diseases [54–56] thanks to their unique chemical nature as segmented polymers containing modifiable soft and hard segments to provide different hydrophobicity, aqueous swelling behaviour, tensile strength, and elongation properties [57]. So far, there has been an increase interest in developing reservoir-type IVRs for sustained delivery of APIs topically at the human FGT [50]. As an attractive IVR design, reservoir IVRs can provide constant drug levels in the FGT over prolonged periods of time [54]. However, due to the manufacturing complexity and cost of reservoir IVRs, matrix IVRs is often the first option of IVR design for researchers [58, 59]. In the recent decade, thanks to advancements in traditional HME technologies and development of advanced extrusion die designs, fabrication of reservoir IVRs with complex structures was achievable [47]. Unfortunately, the high cost of the extrusion and welding equipment as well as the demand of experienced operators and sophisticated die design still create the unavoidable technical barriers to establish such manufacturing capability in a regular laboratory with limited resources. In the current study, we investigated the possibility of introducing easily accessible FDM 3D printing technology for rapid fabrication or reservoir IVRs with different designs. A full-sized macaque reservoir IVR (25 mm outer diameter × 5 mm cross-sectional diameter) can be printed within 15 min and the time required for each HP-60D-35/ATPU-75A segment printing ranged from 5 to 10 min depending on the orientation of the object set on the printing bed and the length/complexity of the segments. In addition, FDM 3D printing significantly shortens the time for reservoir IVR fabrication and it also reduces the cost related to modifying the extrusion dies required for traditional HME. The complex surface porous structures obtained in the present study (Figs. 4–6) were unable to be generated using traditional HME or hot-melt injection molding, making FDM 3D printing a promising technology for producing highly complicated geometries required for drug-eluting devices especially for personalized medicine.

Next, we investigated the impact of FDM 3D printing on the release profiles of different APIs from various generated structures. In the present work, we first examined the different thickness of RCM (also reflected as ro/ri ratios in the current study) produced by FDM 3D printing and its regulation of hydrophilic chemotherapeutic drug in a typical reservoir IVR, since it is the predominant parameter determining drug release rates from end-capped cylindrical reservoir devices when other factors were the same, including length, core drug concentration, and effective diffusivity of the drug through the polymer used for making reservoir IVR [47, 48]. Our results demonstrated that RCM thickness can be simply controlled by changing the number of printing perimeter shells with high reproducibility. A linear correlation (*R*^2^ = 0.99784) of the measured RCM thickness and the printing perimeter was observed (Figs. 2 and 3). The proportionally increased RCM thickness with increased perimeter enabled the fine-tuning of the release rates of HCQ (Fig. 7). Since the daily elution of HCQ from the printed segments was all above the clinically effective HCQ concentration to elicit anti-inflammatory and T-cell immune quiescent activity, the most desired release profile can be assessed and selected in the future via non-human primate models. The prolonged HCQ release through IVRs printed with more than 1 perimeter would also be attractive to develop HCQ IVRs able to deliver for a period of over 30 days. Additionally, to our knowledge, there have not been many studies so far reporting the development of reservoir IVRs to achieve sustained release of hydrophilic APIs. Recent efforts have been made to develop FDM-printed IVRs against vaginal candidiasis or local HIV infections releasing APIs either blended with thermoplastic polyurethanes as a matrix-type IVR [60, 61] or loaded into the lumen similarly like our IVR as a reservoir-type IVR [62]. Arany et al. described a 3D-printed reservoir IVR capable of releasing chloramphenicol and metronidazole at zero-order kinetics but only within 48 h [62]. The release of HCQ from our IVR segments showed tunable zero-order release kinetics for over 14 days, making FDM 3D printing an attractive technology for rapid fabrication of reservoir IVRs to provide controlled sustained delivery of hydrophilic APIs.

To date, there has been limited number of strategies described for the sustained delivery of macromolecules. Radomsky and colleagues described an EVA matrix loaded with fluorescently labeled IgG as well as polysaccharides and salts as pore-forming reagents in the EVA matrix [63] to enable IgG diffusion. A sustainable concentration of 0.3 to 10 µg/mL was maintained in vaginal fluid for 30 days. Recently, two literature reports described unique pod-insertion IVR designs delivering HIV neutralizing antibody [64] and HIV antigen gp140 protein [65] for HIV prophylaxis. Zhao et al. developed a pod-insertion IVR to release a broadly neutralizing antibody for HIV, VRC01-N, up to 21 days in a controlled manner from a compressed solid core (containing trehalose, tween 20, and histidine) through a rate-controlling PLA membrane and 1–1.5 mm diameter delivery pore physically created on the silicone IVR body [64]. The release rates of monoclonal antibody were determined by the antibody solubility, the composition and the thickness of the PLA coating, the size and number of delivery pores, and numbers of pods embedded. Due to the slow erosion of PLA, a zero-order release at the rate of 3.4 mg/day can be maintained in vitro for 10 days with low antibody loading. McKay et al. described another design of pod-insertion IVR using HPMC as the rate-controlling excipient for controlled delivery of recombinant HIV-1 CN54gp140 protein to elicit robust systemic and mucosal humoral immune responses [65]. Each pod was produced by freeze-drying HPMC with the antigen (500 µg) and adjuvant R848 (500 µg) followed by the insertion of the pod into a silicone IVR body. When incubated in VFS, the pods released 88% of the recombinant antigen within 6 h due to the large release pore (2 mm) which opened on the IVR. In the present study, we employed FDM 3D printing to create significantly smaller release pores at mesoscale (200–500 µm) on the reservoir IVR segments (Figs. 4–6) to restrict the diffusion of IgG and small fragment of gp120. Coupled with rate-controlling HPMC, nearly zero-order release kinetics was achieved for both molecules and can be maintained for 2 weeks (Figs. 8 and 9). The uniformly generated mesoscale pores as a result of grid printing pattern and IF settings may contribute to the controlled and sustained protein release. In comparison to the described fabrication process of pod-insertion IVRs, FDM 3D printing demonstrated significant advantage to direct and rapid fabrication of the mesoscale geometries directly on the IVR body, therefore minimizing the separate steps of manufacturing the pod. The diffusion rates of the macromolecules also appear to be related to the size of the molecule as observed in the current study. Small gp120 fragments (2.1 kD) demonstrated a faster release rate compared to IgG (150 kD) (Fig. 9). In addition to protein/peptide delivery using FDM 3D printed segments, we further evaluated its potential to deliver PLGA nanoparticles (Fig. 9). We chose the triangular print pattern generated segments containing larger pore size to release C6NP. Smaller-sized molecules such as IgG and gp120 fragments were suitably released from grid pattern generated segments. C6NP showed significantly slower but controllable release rates, which may be correlated to the different size and shapes of the mesoscale pore formed. Grid printing pattern showed rectangular pores formed on both 50% and 80% IF and more uniformed pore sizes (~ 250 µm pores on 80% IF and ~ 500 µm on 50% IF) with more consistent C6NP diffusion. In contrast, triangular print pattern resulted in the formation of less uniformed pore sizes, resulting in a more fluctuated C6NP release. Prolonged macromolecule and PLGA NP release are preferred to achieve sustained release profile topically within the female genital tract; however, the ideal release rate would need to be selected through in vitro or in vivo efficacy studies in the future. In addition, the 3D printed ATPU-75A segments were non-cytotoxic to vaginal/ectocervical epithelial cells as well as T cells (lack of pro-inflammatory cytokines and chemokines induction), suggesting that FDM 3D printing can be employed to produce biocompatible implantable drug-eluting devices.

The reported IVR systems in the present study enable the possibility to achieve tailored delivery of APIs with different physicochemical properties. Previously, we reported a reservoir type IVR loaded with HCQ that was able to reduce the activation of T cells in a rabbit model [9]. However, more in-depth investigation into potential activity loss or aggregation of temperature and solvent sensitive APIs such as proteins/peptides and NPs during manufacturing and drug release is required.

## Conclusion

Overall, we describe for the first time, the design and fabrication of reservoir-type IVR and IVR segments bearing unique mesoscale structures generated from FDM 3D printing process for controlled delivery of the hydrophilic chemotherapeutic compound HCQ, macromolecules IgG and gp120 peptide, as well as PLGA nanoparticles for extended periods of time. The demonstrated utility of FDM 3D printing to precisely and rapidly fabricate non-cytotoxic IVR segments to achieve tunable release rates and zero-order release kinetics of tested molecules suggest that FDM 3D printing is an attractive manufacturing technology to produce topical drug delivery systems with sophisticated microscale structures.

## Data Availability

All data generated or analyzed during this study are included in this published article.
